# Surgery for severe tricuspid valve regurgitation following blunt thoracic trauma

**DOI:** 10.1186/1749-8090-8-S1-P75

**Published:** 2013-09-11

**Authors:** A Kucuker, M Hidiroglu, H Bayram, M Gokcimen, U Ercelik, E Sener

**Affiliations:** 1 Department of Cardiovascular Surgery, Izmir Katip Celebi University, Ataturk Training and Research Hospital, Karsiyaka-Izmir, Turkey

## Background

Heart valve injuries due to non-penetrating blunt thoracic trauma are rare with aortic valve being the most vulnerable. Tricuspid valve injury resulting in severe regurgitation is unusual after blunt chest trauma. Two major issues for the management of traumatic tricuspid regurgitation are timing and type of the operation. Valve repair is naturally the first choice but valve replacement may be required in severely destructed valves with good surgical outcomes.

## Method

A 61 years old man who suffered a car accident was admitted to emergency department with multiple traumas. He had costal fractures, bilateral pneumothorax and left femur fracture. Chest auscultation revealed 3/6° systolic murmur. Transthoracic echocardiography demonstrated chordal ruptures for anteroseptal leaflet, causing severe tricuspid regurgitation. He was hospitalized in the intensive care unit. Chest tubes were inserted for pneumothorax with orthopedical retraction applied for left femur fracture. He did not develop any symptoms related to severe tricuspid regurgitation and valve surgery was postponed to full recovery of other system injuries. Three months later he was scheduled for elective tricuspid surgery. Median sternotomy and standard aorto-bicaval cannulation was performed. Tricuspid valve was severely destructed with multiple leaflet lacerations, chordae and papillary muscle ruptures. Tricuspid repair was not possible and valve replacement with 31mm bioprosthesis (Aspire, Vascutek-Terumo, USA) was performed.

## Results

Intensive care unit and subsequent ward follow up was uneventful. He was discharged at postoperative 6th day. His nine months follow up revealed an asymptomatic patient with a functional capacity of NYHA 1.

## Conclusion

Although rare, heart valve injuries may occur following blunt thoracic trauma. Transthoracic echocardiography is a must for evaluating such patients. Surgery if necessary can then be planned either urgent or electively according to each patient situation.

